# Different Toll-Like Receptor Stimuli Have a Profound Impact on Cytokines Required to Break Tolerance and Induce Autoimmunity

**DOI:** 10.1371/journal.pone.0023940

**Published:** 2011-09-12

**Authors:** Albert C. C. Lin, Dilan Dissanayake, Salim Dhanji, Alisha R. Elford, Pamela S. Ohashi

**Affiliations:** 1 Campbell Family Institute for Breast Cancer Research, Ontario Cancer Institute, Toronto, Ontario, Canada; 2 Department of Immunology, University of Toronto, Toronto, Ontario, Canada; 3 Department of Medical Biophysics, University of Toronto, Toronto, Ontario, Canada; INRA, France

## Abstract

Although toll-like receptor (TLR) signals are critical for promoting antigen presenting cell maturation, it remains unclear how stimulation via different TLRs influence dendritic cell (DC) function and the subsequent adaptive response in vivo. Furthermore, the relationship between TLR-induced cytokine production by DCs and the consequences on the induction of a functional immune response is not clear. We have established a murine model to examine whether TLR3 or TLR4 mediated DC maturation has an impact on the cytokines required to break tolerance and induce T-cell-mediated autoimmunity. Our study demonstrates that IL-12 is not absolutely required for the induction of a CD8 T-cell-mediated tissue specific immune response, but rather the requirement for IL-12 is determined by the stimuli used to mature the DCs. Furthermore, we found that IFNα is a critical pathogenic component of the cytokine milieu that circumvents the requirement for IL-12 in the induction of autoimmunity. These studies illustrate how different TLR stimuli have an impact on DC function and the induction of immunity.

## Introduction

Since the discovery of toll-like receptors (TLRs), we have come to appreciate their crucial role in the induction of adaptive immunity against pathogens. In response to microbes, the engagement of pathogen associated molecular patterns by TLRs initiates a complex process of antigen presenting cell (APC) maturation and pro-inflammatory cytokine production [1], [2]. These intricately coordinated cellular processes are instrumental to the functional differentiation of pathogen-specific T cells. Although it is suggested that stimulation via different TLRs is sufficient to promote distinct immune responses, very little is known about how this program is set by mature DCs. This issue is particularly difficult to dissect *in vivo*, since it's not possible to directly compare stepwise events that occur upon bacterial versus viral infections.

Previous studies have shown that the maturation of DCs is a key event that promotes autoimmunity in a variety of models [3], [4], [5]. Evidence indicates that, TLR3 and TLR7 stimulation are critical for the activation and recruitment of autoreactive CD8^+^ T cells and subsequent destruction of pancreatic islet β cells [6]. Activation of APCs via TLR4 or TLR9 can also disrupt self-tolerance and result in the induction of EAE [7]. Likewise, TLR3, TLR4 and TLR9 appear to play a critical role in the development of autoimmune myocarditis [8], [9]. While infection is a well recognized trigger of autoimmunity, recent studies suggest that endogenous TLR ligands expand TLR signaling capacity and may therefore play a role in autoimmune disorders including those arising from sterile inflammation [10].

Stimulation of different PRR has the potential to induce a particular cocktail of pro-inflammatory cytokines. A given cytokine milieu may then direct the initiation of distinct adaptive immune responses. Hirschfeld et al. along with subsequent studies shed light on the cytokine specificity of TLR2 and TLR4 and its impact on T helper cell differentiation [11], [12]. Following TLR4 engagement by lipopolysaccharide (LPS), murine macrophages produce large amounts of tumor necrosis factor alpha (TNFα), interleukin (IL)-1β, IL-12 and IP-10 which selectively induced a Th1 response. In contrast, peptidoglycan (PGN) engagement of TLR2 induces moderate production of TNFα and IL-1β, without IL-12 and IP-10, biasing toward a Th2 response [11], [12], [13].

Evidence suggests that differential adaptor engagement and downstream signaling by different TLRs can contribute to the production of a given cytokine milieu. For instance, TLR3 and TLR4 can signal via TRIF and activate IRF3 dependent production of IFNβ [14], [15]. TLR sensors of nucleic acids, TLR3, TLR7, TLR8 and TLR9, have the ability of producing both IFNα and IFNβ via IRF3 and IRF7 activation [15], [16]. In addition to differential adaptor engagement and downstream signaling, qualitative parameters of the TLR ligand interaction itself may contribute to the induction of certain cytokines, as in the case of TLR9 signaling where distinct endosomal trafficking of A type but not B type CpG result in IRF-7 and IFNα production [17], [18].

One key cytokine that is produced upon DC maturation is the pro-inflammatory cytokine IL-12 [19]. IL-12, a heterodimeric cytokine consisting of the IL-12p35 and IL-12p40 subunits, is readily produced by stimuli such as double stranded RNA, LPS, flagellin, single stranded RNA and bacterial DNA [20]. Although evidence suggests that IL-12 plays an important role and has been referred to as signal 3, after the TCR induced signal 1 and costimulatory signal 2 [21]. The role of IL-12 in the induction of adaptive immune responses remain controversial [22]. In addition to playing a significant role in the induction of Th1 responses, IL-12 also the functional maturation of cytotoxic T lymphocytes (CTLs) by augmenting proliferation, survival and generation of effector molecules such as perforin and granzymes [23]. Studies have also identified IL-12's role in modulating lymphocyte trafficking by modulating P-SGL1 expression [24].

Given IL-12's involvement in promoting multiple facets of T cell immunity, it is believed to be a key mediator of autoimmunity and has been widely used as a marker for DC activation in cancer immunotherapy [25], [26]. Indeed, studies in several murine experimental models and clinical settings also point to IL-12 playing an important role in the development organ specific autoimmunity. Analysis in autoimmune prone murine strains demonstrated that predisposition to autoimmunity may be attributed to enhanced IL-12 production by APCs due to alterations in the induction of NF-κB [27], [28]. In an autoimmune myocarditis model, IL-12 facilitates the differentiation of pathogenic CD8 T cell effectors [29]. In the non-obese diabetic (NOD) model, the administration of IL-12 or ablation of IL-12 mediated Stat4 signaling can markedly accelerate or completely prevent the onset of diabetes respectively [30], [31]. Furthermore, blocking IL-12 in patients with active Crohn disease, which is commonly associated with increased production of IL-12 by APCs, can induce stable remission [32].

IL-12's impact on the development of organ specific autoimmunity is however not decidedly pathogenic across all experimental settings. In some models, IL-12 appears non-pathogenic or even protective [33], [34]. Complicating the interpretation of these data is the discovery of the anti-inflammatory effects of closely related cytokine IL-35, which has redundant usage of the IL-12p35 subunit [35]. One potential contributing factor to IL-12's divergent role in the pathogenesis of autoimmune disorders may be related to the different molecular mechanisms that are necessary to initiate different autoimmune diseases. It is possible that in certain models of autoimmunity, the induction of disease depends on certain cytokines induced by different PRR ligands.

The current study investigated whether different APC maturation stimuli influenced the requirement for IL-12 in the induction of a CD8 T cell mediated autoimmune response *in vivo*. In this report, we used the previously characterized RIP-gp transgenic model, where the lymphocytic choriomeningitis virus glycoprotein (LCMV-GP) is expressed in the pancreatic islet β cells [36]. In this model, T cells specific for the LCMV-GP remain ignorant towards the LCMV-GP expressed in the pancreas under the steady state. Upon activation, these autoreactive CD8^+^ T cells infiltrate the pancreatic islets and mediate the destruction of the GP+ β-cells, resulting in diabetes. Recent work has shown an alternate way to activate endogenous GP-specific T cells using mature bone marrow derived dendritic cells (BMDCs). We have compared the ability of Poly I∶C and LPS to mature DCs since TLR3 acts via the adapter TRIF, while TLR4 uses both TRIF and MyD88. By maturing WT or IL-12 deficient BMDCs with Poly I∶C or LPS and pulsing mature DCs with GP peptides, the impact of TLR3 or TLR4 stimuli on the requirement for IL-12 in breaking tolerance and inducing autoimmunity can be evaluated. Unexpectedly our studies showed that autoimmunity induced with Poly I∶C matured DCs is IL-12 independent, whereas IL-12 was essential for the induction of autoimmunity using LPS matured DCs. Our analysis also showed that Poly I∶C stimulated DCs produce high amounts of IFNα, compared with LPS stimulated DCs. Accordingly, IFNα given together with LPS stimulated DCs was able to induce autoimmunity in the absence of IL-12. Therefore, IFNα can overcome the requirement for IL-12 in the induction of T cell mediated pathology elicited by LPS-matured DCs. These studies clearly demonstrate that different TLR matured DCs require different cytokines to elicit a functional immunopathological adaptive immune response. These observations have important implications for controlling tissue specific autoimmunity and anti-tumor immunity.

## Results

### TLR induced DC maturation programs the requirement for IL-12 in the induction of autoimmunity

**Figure 1:**
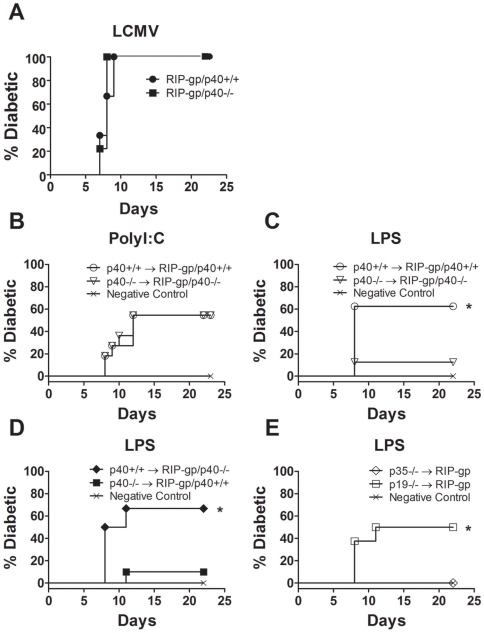
APC stimulation confers differential requirement of IL-12 in the induction of autoimmunity. (A) Diabetes incidence in RIP-gp/p40+/+ (solid circle) and RIP-gp/p40−/− (solid square) mice that were infected with LCMV-WE on day 0. Results are from 9 mice per group, 2 independent experiments. (B, C) Diabetes incidence in RIP-gp/p40+/+ (open circle) and RIP-gp/p40−/− (open triangle) mice treated with p40+/+ and p40−/− BMDCs respectively, pulsed with peptides derived from LCMV-GP and stimulated with (B) LPS or (C) Poly I∶C. (D) Diabetes incidence in RIP-gp/p40+/+ (solid square) and RIP-gp/p40−/− (solid diamond) mice treated with p40−/− and p40+/+ BMDCs respectively, pulsed with peptides derived from LCMV-GP and stimulated with LPS. (E) Diabetes incidence in RIP-gp mice treated with IL-12p35−/− (open diamond) and IL-12p19−/− (open square) BMDCs pulsed with peptides derived from LCMV-GP and stimulated with LPS. In each experiment, non-peptide pulsed DCs were included as negative controls (Cross). Results in (B) to (E) are from 7 to 11 mice per group, 2 independent experiments. A test of statistical significance of p<0.05 by the Mantel-Cox Test and Gehan-Breslow-Wilcoxon Test is denoted with * in (B), (D) and (E).

We first explored whether the p40 subunit of IL-12 plays an obligatory role in the induction of autoimmunity in our model. RIP-gp/p40−/− mice were generated and infected with LCMV and blood glucose was monitored for 3 weeks. Approximately 8 to 10 days after LCMV infection, all LCMV-infected RIP-gp/p40−/− mice were diabetic. There was no significant difference in the kinetics or incidence of disease in comparison with LCMV-infected RIP-gp/p40+/+ mice (Figure 1A). Therefore, the p40 subunit of IL-12 does not appear to play an essential role in virus induced autoimmunity.

We further evaluated the role of the p40 subunit using an alternate method to induce autoimmunity. BMDCs were matured using Poly I∶C or LPS, then pulsed with both class I and class II LCMV-GP peptides (gp33, gp276, and gp61 peptides) and given i.v. to RIP-gp transgenic mice. Poly I∶C matured p40−/− BMDCs were as effective as wild type BMDCs in inducing diabetes (Figure 1B). However, the treatment of RIP-gp/p40−/− mice with LPS matured p40−/−BMDCs showed a reduced incidence of diabetes, in contrast to the treatment of RIP-gp/p40+/+ mice with LPS-matured p40+/+ BMDCs (Figure 1C). The induction of diabetes using this DC based model is dependent upon CD8 T cells (Dissanayake D. unpublished). Therefore, the absolute requirement for the p40 subunit of IL-12 is dependent upon the stimuli that triggered DC maturation.

Since activated B cells, macrophages, and neutrophils express p40 [19] it remains possible that the requirement for p40 in the induction diabetes may reflect a defect in B cell, macrophage, or neutrophil functions. To determine whether the ability of the DC alone to produce p40 could influence diabetes induction, we treated RIP-gp/p40+/+ mice with LPS matured p40−/− BMDCs or conversely treated RIP-gp/p40−/− mice with LPS matured p40+/+ BMDCs (Figure 1D). RIP-gp/p40−/− mice treated with LPS matured p40+/+ BMDCs developed diabetes, but not vice versa clearly demonstrating that p40 production is required by LPS matured DCs to induce CD8 mediated immune pathology.

Studies have shown that the IL-12p40 subunit can also heterodimerize with the p19 subunit to form IL-23 [37]. IL-23 plays an important pathogenic role in several autoimmune models via the induction and maintenance of Th17 cells [33], [34]. To determine whether the lack of diabetes induction can be attributed to the lack of IL-23, we treated RIP-gp mice with LPS matured p35−/− BMDCs or p19−/− BMDCs. Fifty percent of RIP-gp mice treated with LPS matured p19−/− BMDCs developed diabetes, unlike the mice given LPS matured p35−/− BMDCs, indicating that the production of IL-12 by DCs is critically important for diabetes induction (Figure 1E). Together, our data demonstrate that the different pathogen-related stimuli define the requirement for IL-12 in the induction of autoimmunity. Specifically, IL-12 production by DCs plays a critical role in diabetes induction when DCs are matured with LPS, but not with Poly I∶C treatment or LCMV infection.

### Reduced T cell infiltration in mice treated with LPS matured DCs

**Figure 2:**
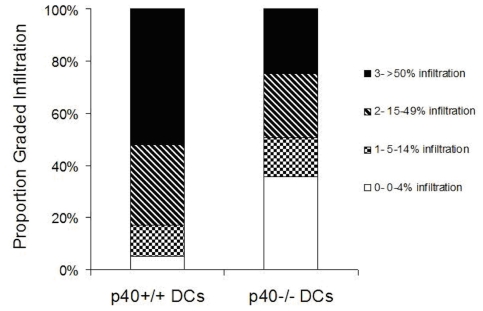
Limited CD8 infiltration in mice treated with LPS matured DCs. Degree of CD8+ islet infiltration in RIP-gp mice treated with LPS stimulated peptide-pulsed p40+/+ or p40−/− BMDCs. Results are from a minimum of 5 mice per group, 100 islets per group from 2 independent experiments.

To gain insights into the mechanism through which LPS matured DCs have a limited capacity to induce diabetes, the pancreas was evaluated by immunohistochemistry. Five days after RIP-GP mice were given WT or p40−/− LPS matured DCs, the pancreas was taken and stained for CD8 T cell infiltration (Figure 2). RIP-GP mice that were given LPS treated p40−/− DCs showed reduced CD8+ T cell inflammation in the islets which correlated with a reduced incidence of diabetes. A series of experiments were done to evaluate why there may be reduced CD8 inflammation.

### Induction of T cell responses in the absence of p40

**Figure 3:**
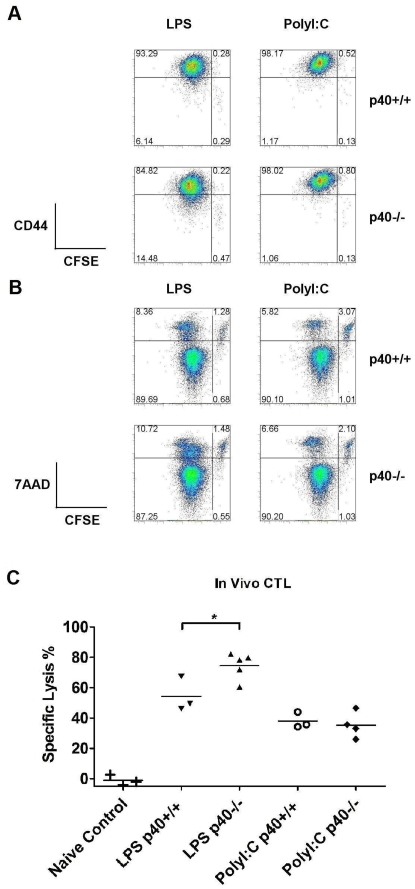
IL-12 is not required for CD8 T cell proliferation, survival, activation and CTL differentiation. (A, B) CFSE-labeled P14 transgenic T cells were cultured in media alone or co-cultured with either p40+/+ or p40−/− BMDCs stimulated with LPS or Poly I∶C and pre-pulsed with gp33 peptides. Cultures were assessed by flow cytometry 3 days later. Representative plots of CFSE dilution, CD44 (A) and 7AAD (B) staining on gated CD8+ cell populations and the percentages of cells in each quadrant are displayed. Results are representative of 3 independent experiments of 2–3 mice per group. (C) *In vivo* CTL activity of C57BL/6 mice treated with PBS (naïve control) or administered with p40+/+ and p40−/− BMDCs pulsed with gp33 peptide and stimulated with LPS was assessed on day 5 by flow cytometry analysis of the remaining ratio of gp33 peptide pulsed splenocytes to negative control AV peptide pulsed splenocytes given i.v. 4 hours prior. Results are representative of 2 independent experiments of 3–5 mice per group. A test of statistical significance of p<0.05 by the Student T-Test is denoted with *.

IL-12 has previously been described to be important for T cell survival and the induction of effector functions [38]. Therefore, we examined the impact of IL-12 on LCMV-GP specific T cell responses. CFSE labeled P14 T cells specific for gp33 peptide in the context of H-2^b^ [39] were stimulated with gp33 peptide pulsed, LPS or Poly I∶C matured p40+/+ and p40−/− BMDCs for 3 days *in vitro*. In the absence of IL-12 secretion by DCs, antigen-specific T cell proliferation and upregulation of the activation marker CD44 was unaffected (Figure 3A). Furthermore, the lack of IL-12 did not enhance T cell apoptosis since the proportion of CD8 T cells that were CFSE-7AAD+ were comparable between co-cultures with p40+/+ and p40−/− BMDCs under all stimulation conditions (Figure 3B).

To determine whether T cell functional maturation was impaired in the absence of IL-12, we evaluated cytotoxic activity in DC primed mice. C57BL/6 mice were treated with LPS or Poly I∶C stimulated p40+/+ and p40−/− BMDCs pulsed with gp33 peptide. gp33 specific cytolytic activity was assessed *in vivo* on day 5 by quantifying the ratio of gp33 peptide pulsed targets to AV peptide pulsed control target cells remaining in the spleen of primed C57BL/6 mice. C57BL/6 mice given Poly I∶C stimulated p40−/− BMDCs showed gp33-specific cytolytic activity comparable to C57BL/6 mice treated with Poly I∶C stimulated p40+/+ BMDCs (Figure 3C). Interestingly, C57BL/6 mice given LPS stimulated p40−/− BMDCs had higher gp33 specific cytolytic activity compared with C57BL/6 mice treated with LPS stimulated p40+/+ BMDCs (Figure 3C). Therefore changes in CD8+ T cell activation, function or survival was unlikely to account for differences observed in the infiltration of the pancreas of mice treated with LPS matured p40−/− DCs.

### Evaluating DC cytokine production in the absence of p40

**Figure 4:**
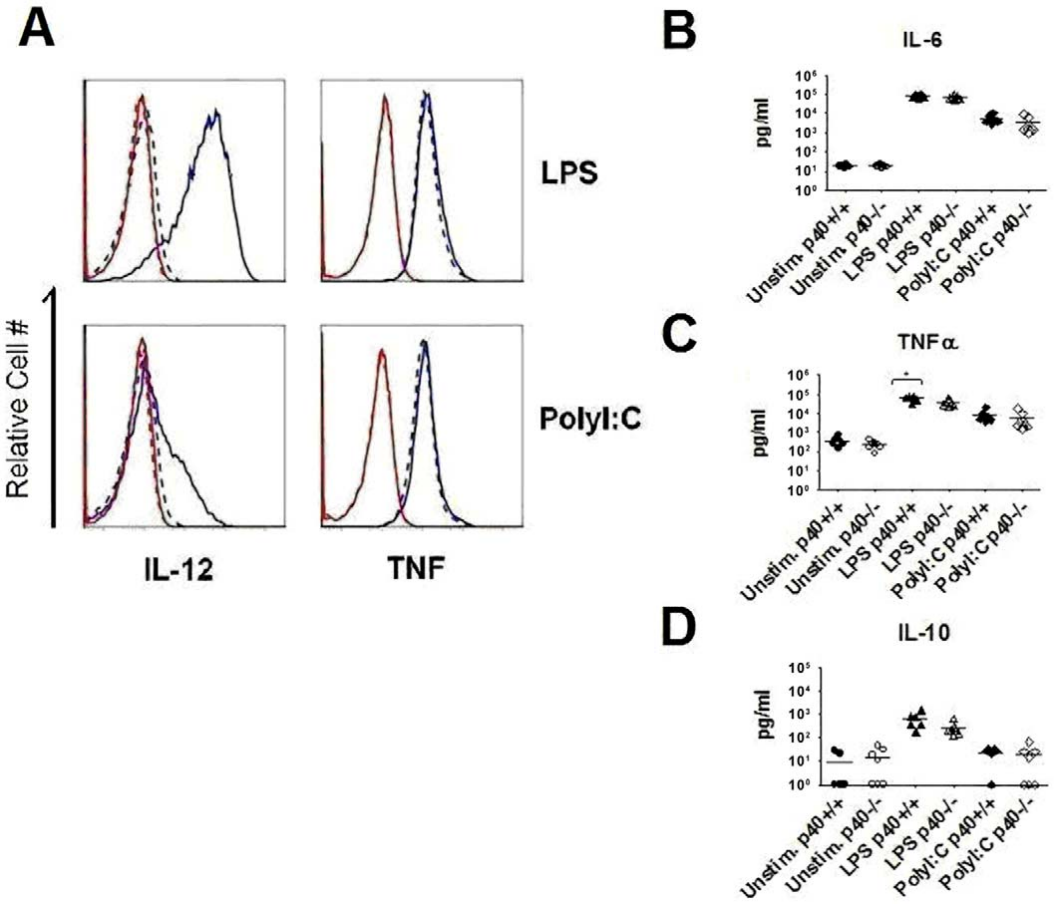
Cytokine profiles of BMDCs remain largely unaltered in the absence of IL-12. (A) Expression of IL-12 and TNFα assessed by intracellular cytokine staining. p40+/+ and p40−/− BMDCs were stimulated with LPS, PolyI∶C or left unstimulated for 12 hours and assessed by flow cytometry. Representative IL-12 and TNFα profiles on gated CD11c+CD11b+ cell populations are presented with p40+/+ shown in blue solid lines and p40−/− shown in blue dashed lines. Isotype controls for p40+/+ and p40−/− are displayed in red solid and dashed lines respectively. Results are representative of 3 independent experiments of 2–3 mice per group. (B, C, D) Cultured supernatants of p40+/+ and p40−/− BMDCs stimulated with LPS, PolyI∶C or left unstimulated for overnight were assessed by Cytometric Bead Array. Results are from 2 independent experiments with a minimum of 6 mice per treatment group. A test of statistical significance of p<0.05 by the Student T-Test is denoted with *.

Studies have shown that the induction of pro-inflammatory mediators by DCs plays a critical role in promoting the maturation and homing of T cells [4]. To determine whether the induction of autoimmunity by LPS or Poly I∶C matured DCs was linked to the production of different cytokines, we compared the cytokine production from p40+/+ and p40−/− BMDC's. 12 hours after stimulation with LPS, p40+/+ BMDCs exhibit robust production of IL-12 (Figure 4A). Although Poly I∶C induced IL-12 production, the response is clearly less than that observed after LPS stimulation. Notably, the induction of intracellular levels of TNFα was similar upon LPS versus Poly I∶C stimulation. Therefore IL-12 is differentially induced in LPS matured DCs compared with Poly I∶C maturation, and IL-12 was essential for the induction of autoimmunity in our model after LPS -induced DC maturation.

Cytokine production was also evaluated in the supernatant from these cultures using a cytometric bead assay. p40+/+ and p40−/− BMDCs stimulated with LPS or Poly I∶C showed an approximate 10,000 fold and 1,000 fold increase in the production of IL-6 compared to unstimulated control cultures respectively, but revealed no significant difference in the production of IL-6 in the absence of p40 (Figure 4B). P40+/+ and p40−/− BMDCs stimulated with LPS and Poly I∶C induced an approximate 200 fold and 20 fold increase in the production of TNFα respectively (Figure 4C). In the absence of IL-12, LPS stimulated p40−/− BMDCs had a slight reduction in TNFα production detectable by the cytometric bead assay that was not observed by intracellular cytokine staining (Figure 4C and A, T-test p<0.05). No difference in TNFα production was observed between poly I∶C stimulated WT and p40−/− DCs. We also examined the production of the anti-inflammatory cytokine IL-10 and chemotactic factor MCP-1 (data not shown) but did not find a significance difference in production with either stimulation conditions in the presence or absence of IL-12. Similar to observations by others, there appears to be a qualitative difference in the ability of LPS and Poly I∶C to induce IL-10 production, as only LPS stimulation induced significant IL-10 production (Figure 4D) [40]. Although these studies were done to examine whether the absence of p40 would have a negative impact on cytokine production, we found that the intrinsic ability of LPS versus Poly I∶C to stimulate cytokines had more influence on the levels of cytokines that were produced, than the absence of p40.

### IFNα can promote autoimmunity in the absence of IL-12

**Figure 5:**
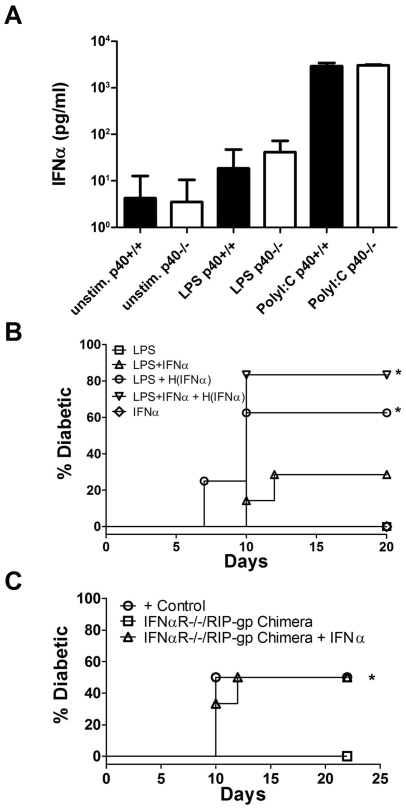
Exogenous IFNα can overcome the absence of DC-derived IL-12 in the induction of autoimmunity. (A) IFNα production was measured in the supernatant from unstimulated, LPS stimulated and PolyI∶C stimulated p40+/+ and p40−/− BMDCs by ELISA. Results are from 4 mice per treatment group and representative of 3 independent experiments. (B) Diabetes incidence in RIP-gp mice treated with IFNα alone (open diamond), peptide-pulsed p40−/− BMDCs stimulated with LPS (open square) or peptide-pulsed p40−/− BMDCs stimulated with LPS plus IFNα (open triangle). Injection of 10000 u of IFNα to RIP-gp mice 3 days after treatment with LPS stimulated peptide-pulsed 40−/− BMDCs or LPS and IFNα stimulated peptide-pulsed p40−/− BMDCs (open circle and open upside-down triangle respectively). (C) Diabetes incidence in IFNαR+/+/RIP-gp bone marrow chimeras treated with LPS stimulated p40+/+ BMDCs (open circle) and IFNαR−/−/RIP-gp chimeras treated with LPS stimulated p40−/− BMDCs (open square) or an additional i.v. injection of IFNα 3 days later (open triangle). A test of statistical significance of p<0.05 against RIP-gp mice (B) and IFNαR−/−/RIP-gp mice (C) treated with p40−/− BMDCs stimulated with LPS is denoted with *. Results shown are from a minimum of 6 mice per group from 2 independent experiments.

Since the absence of p40 did not result in a profound reduction in cytokine secretion by BMDCs, we decided to evaluate whether different levels of cytokines were made upon LPS versus Poly I∶C stimulation of BMDCs. It is possible that cytokines that are produced by DCs after Poly I∶C stimulation, are able to bypass the requirement for IL-12. One significant qualitative difference noted in the literature between TLR4 and TLR3 stimulation is the robust induction of IFNα by TLR3 [41]. Considering IFNα has been described to negatively regulate IL-12 production [42] and our findings that diabetes induction is independent of IL-12 when autoreactive T cells are activated when IFNα is significantly represented in the cytokine milieu (with LCMV infection or Poly I∶C stimulation), the possibility arises that IFNα induction is critical and may overcome the requirement for IL-12 in LPS matured DCs. Indeed, in characterizing the IFNα response in our model, we observed robust production of IFNα, 12 hours after stimulating BMDCs with Poly I∶C but not LPS (Figure 5A). However, we found no evidence of reciprocal co-regulation of IFNα by IL-12 as IFNα production was not significantly enhanced in the absence of IL-12. Therefore, it is clear that Poly I∶C stimulation leads to high levels of IFNα production that is independent of p40 in our culture conditions, and thus IFNα may have a critical role in promoting autoimmunity in this model.

To investigate whether IFNα can overcome the requirement for IL-12 in LPS matured DCs, RIP-gp mice were given LPS matured p40−/− DCs with various combinations of IFNα. IFNα was included in BMDC cultures to promote DC maturation and/or administered *in vivo* 3 days after DC treatment, to evaluate the impact on the induction of diabetes. The following combinations were tested: LPS matured p40−/−DCs, LPS plus IFNα matured p40−/−DCs, LPS matured p40−/−DCs with IFNα treatment 3 days later, or LPS plus IFNα matured p40−/−DCs with IFNα treatment 3 days later (Figure 5B). As in previous experiments, RIP-gp/p40+/+ hosts given LPS matured p40−/−DCs did not lead to the induction of autoimmunity. Although nearly a quarter of RIP-gp/p40+/+ hosts receiving LPS plus IFNα matured p40−/−DCs became diabetic, the effect of IFNα on diabetes induction had the most impact when RIP-gp/p40+/+ mice were given IFNα 3 days after treatment with LPS matured or LPS plus IFNα matured p40−/−DC (Figure 5B). Treatment of RIP/p40+/+ mice with IFNα alone was not sufficient to induce diabetes. These results suggest that IFNα is a critical cytokine that can circumvent the requirement for IL-12 and therefore is a key mediator of autoimmune pathology in the absence of IL-12. Furthermore, our results suggest that the effects of IFNα's may be mediated by modifying the host's T cells or target organ in addition to directly enhancing the immunostimulatory activities of IL-12 deficient DCs.

IFNα has previously been described to enhance the effector functions and survival of CD8 T cells [23], [38], [43], [44]. To assess whether IFNα circumvents the LPS specific requirement for IL-12 by enhancing host's T cell responses, we examined the *in vivo* CTL activity of p40+/+ C57BL/6 primed with LPS plus IFNα stimulated peptide pulsed p40−/− BMDCs or LPS stimulated p40−/− BMDCs followed by the administration of exogenous IFNα to the host 3 days later. However, we did not observe a change in gp33 specific CTL activity in either treatment condition (data not shown). Furthermore, in lethally irradiated RIP-gp mice reconstituted with IFNαR−/− bone marrow, treatment with LPS stimulated p40−/− BMDCs followed by the administration of IFNα 3 days later induced autoimmunity with similar kinetics and incidence of disease compared to RIP-GP/p40+/+ mice reconstituted with IFNαR+/+ bone marrow (Figure 5C). These data indicate that exogenous IFNα does not act directly on host T cells or on secondary events involving DC populations (such as cross priming) and acts by promoting inflammation.

**Figure 6:**
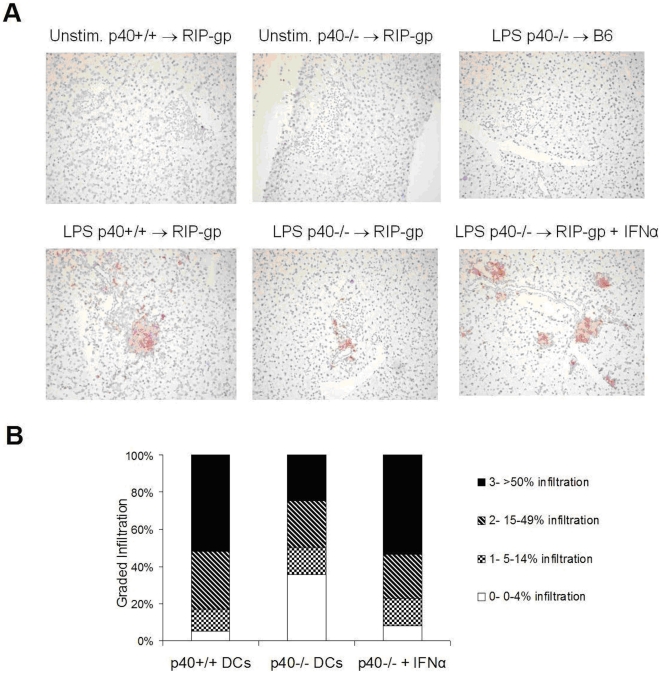
Exogenous IFNα enhances CD8 infiltration. (A) Insulitis as assessed by immunohistochemistry of CD8 infiltrates (stained red) in pancreas sections five days after treatment. Representative sections from RIP-gp mice treated with p40+/+ (bottom left panel), p40−/− (bottom middle panel) peptide-pulsed BMDCs stimulated with LPS or p40−/− peptide-pulsed BMDCs stimulated with LPS with an additional i.v. injection of IFNα (bottom right panel) are displayed. For controls, representative sections of RIP-gp mice treated with unstimulated peptide-pulsed p40+/+ (top left panel) or p40−/− (top middle panel) BMDCs and C57BL/6 mice treated with LPS stimulated peptide-pulsed p40−/− BMDCs (top right panel) are shown. (B) Quantiation of CD8 infiltration, with the first 2 columns represented in figure 2 but reproduced here for comparison. Results are representative of a minimum of 5 mice per group, 100 islets per group from 2 independent experiments.

To determine whether IFNα plays a role in promoting an inflammatory response in the absence of IL-12, RIP-gp hosts were given LPS matured p40+/+ BMDCs or LPS matured p40−/− BMDCs and CD8+ T cell infiltration in the pancreas was examined five days post-treatment by immunohistochemistry. In the absence of IL-12, RIP-gp mice treated with LPS matured p40−/− BMDCs have clearly reduced CD8 T cell infiltration in the pancreatic islets (Figure 2, 6). The CD8 T cell infiltration was both antigen specific and dependent on the activation of BMDCs as infiltration was not observed in C57BL/6 mice treated with LPS matured p40+/+ BMDCs (no peptide) or in RIP-gp mice given immature peptide pulsed p40−/− or p40+/+ BMDCs (Figure 6). Furthermore, when RIP-gp mice were treated with exogenous IFNα 3 days after injection with LPS matured p40−/− BMDCs, autoreactive CD8 T cell infiltration was enhanced and comparable to that observed in RIP-gp mice injected with LPS matured p40+/+ BMDCs (Figure 6). Quantitative analysis of the degree of infiltration showed that that percent of islets with heavy infiltration is similar to mice immunized with WT BMDCs (Figure 6B). Together, our results indicate that IFNα can compensate for IL-12's important role in diabetes induced by LPS matured DCs by promoting CD8+ T cell infiltration.

## Discussion

With this model, we can evaluate the impact of different TLR signals on the induction of adaptive immunity *in vivo*, using the same population of DCs and the same defined antigens. We have uncovered novel insights for how different TLR maturation signals influence the induction of autoimmunity. We demonstrated that the transfer of TLR3 or TLR4 matured DCs presenting self-antigens promotes CD8 T cell mediated autoimmune responses and overt organ specific autoimmunity *in vivo*. Our findings add to the growing body of evidence implicating a wide breadth of TLRs in the pathogenesis of autoimmune disorders and support the rationale behind targeting TLRs to promote tumor specific immune responses [45]. Our investigation also revealed previously unappreciated dynamics between TLR signaling and their pro-inflammatory mediators in the pathogenesis of autoimmunity. Specifically, our findings highlight the crucial influence of the type of stimuli used to promote DC maturation and the differential requirement for IL-12 in the pathogenesis of autoimmunity *in vivo*.

### Importance of IL-12 in the induction of autoimmunity is dependent upon TLR stimulation

Within the context of the dialogue between APCs and naïve T cells, along with antigen and co-stimulation, IL-12 produced by APCs is thought to play a crucial instructive role in determining the cellular fate of naïve T cells and has thus been coined the term “Signal 3” [23], [38]. In contrast to this work, our study has shown IL-12 surprisingly plays little role in mediating the proliferation, activation or survival of CD8 T cells. Furthermore, IL-12 appears to have minimal impact on the functional differentiation of CTLs *in vivo*. Our findings suggest a rather surprisingly minimal physiological role for IL-12 in these facets of CD8 T cell functional maturation *in vivo*.

In spite of the normal induction of CTLs after stimulation *in vivo* with p40 deficient DCs, TLR4 mediated induction of autoimmune diabetes is completely abrogated in the absence of IL-12. An examination of the severity of inflammation in the pancreatic β islets cells indicated that IL-12 is an important component of the inflammatory milieu induced by TLR4 signaling, and promotes CD8 T cell inflammation. Previous studies have found IL-12 to be a critical pathogenic factor in promoting local inflammation and diabetes [46]. In addition, during the priming stage of naïve T cells, IL-12 may play a direct or indirect role, i.e. via regulating the expression of co-stimulatory molecules, in modulating the expression of homing and trafficking molecules by CD8 T cells [24]. As our experiments are done in p40 sufficient RIP-gp hosts, our findings suggest DC-produced IL-12 plays an important role in programming CD8 T cell trafficking during early encounter with cognate antigen bearing DCs.

In stark contrast to the importance of IL-12 in TLR4 mediated DC maturation and subsequent induction of diabetes, IL-12 was not required in TLR3 mediated DC induction of autoimmunity. IL-12 was not an essential component of the cytokine milieu induced after TLR3- induced DC maturation and was not required to facilitate CD8 T cell proliferation, survival, functional maturation and homing to target tissues. The emerging paradigm from studies with infectious agents suggests that IL-12 plays a pivotal role in promoting T cell responses to bacterial and parasitic infections, while it is not required in some viral infections [47], [48], [49], [50]. Our model provides a direct way to evaluate the differences in bacterial induced DC maturation (LPS) versus virus induced DC maturation (Poly I∶C). It is not possible to directly compare the induction of immunity by various pathogens because they may have different inherent ways to mature the DC *in vivo*. Furthermore, these pathogens will present a multitude of antigens that are presented in different contexts and different doses. Using our model, we can directly compare the *in vivo* consequence of LPS versus Poly I∶C mature DCs using the same antigens. Our data directly demonstrate that IL-12 is required for the induction of autoimmunity using LPS matured DCs but not Poly I∶C matured DCs, and support the hypothesis that IL-12 is required for bacterial but not viral infections.

### Predominant role for IFNα in promoting tissue specific T cell responses

In order to explain why TLR 3 was not dependent upon IL-12 producing DCs to induce CD8+ T cell mediated pathology, we hypothesized that other components of the inflammatory milieu induced by TLR3 stimulation may functionally compensate for IL-12. Although we found no conclusive evidence of compensatory upregulation of proinflammatory mediators such as TNFα, IL-6 and MCP-1, or downregulation of anti-inflammatory cytokines such as IL-10, we detected a clear quantitative difference in IFNα production between TLR3 and TLR4 stimulated BMDCs. Previous studies have suggested that there may be functional redundancy between IL-12 and IFNα/β signaling in facilitating T cell responses against some viral infections [51]. Other studies have shown that IFNα but not IL-12 p40 subunit is critical for the induction of CD4+ Th1 responses after systemic administration of Poly I∶C [52]. Using this model, our results indicate that in the absence of IL-12, IFNα is a critical pathogenic mediator of autoimmunity *in vivo*.

In contrast with previous studies which have shown IFNα directly enhances T cell survival and effector functions [23], [38], [43], [44], IFNα does not act directly on T cells to mediate CD8 T cell mediated autoimmune responses in our model (Figure 5C). Neither did we find evidence of enhanced CTL activity in mice treated with exogenous IFNα. In SLE patients, the heightened level of IFNα in their sera is thought to contribute to the breakdown of peripheral tolerance via the induction of DC differentiation [53], [54]. In contrast, studies have shown that DC treatment with proinflammatory cytokines are not sufficient to promote functional T helper immunity [55], while other studies have shown that Poly I∶C treatment can promote autoimmunity, only when islet cells are engineered to express CD80 [56]. Our results suggest IL-12 and IFNα both act to promote CD8 T cell trafficking and local inflammation to the target organ. This is supported by previous observations of IFNα's critical role in autoimmunity by promoting inflammation and upregulating MHC class I molecules on the target tissues [6], [57]. It is likely that IFNα influences immunity in multiple ways in different models because of the variety of ways it has been shown to impact the immune response.

Although we have not examined the relative requirements for IFNα for the induction of diabetes in this model by performing the reciprocal experiments with IFNα deficient DCs matured in different activation contexts, studies suggest that IFNα is likely a required factor in the context of TLR activation associated with viral infections [6], [58]. However, the absolute requirement for IFNα production by DCs matured with different TLR stimuli has yet to be elucidated in our model.

### Perspectives

In the classical paradigm of DC-T cell interaction, DCs exist in 2 basic functional states, immature and mature. Naïve T cells that encounter antigen on immature DCs become tolerized while those that encounter antigen on mature DCs become activated. However, current evidence extends this notion and suggests that DCs exist in ‘states’ other than simply immature or mature and that these states can be modified by other factors or in some cases other subsets of cells [59], [60], [61], [62]. One would predict that maturation with different pattern recognition motifs should induce a different type of DC and a corresponding pathogen related immune response. Our findings begin to unravel some of these intricacies, and using the same peptides and same DC subset, show that LPS matured DCs are functionally different from Poly I∶C matured DCs in their ability to induce autoimmunity *in vivo*. Furthermore, our studies suggest that IL-12 may not be an appropriate surrogate marker for functionally mature DCs and the relevance of using IL-12 to extrapolate immunogenicity of DC vaccines in tumor immunotherapy should be reconsidered [63]. Furthermore, therapeutic targeting of IL-12 for treatment of autoimmunity should also take into consideration the potential importance of IFNα in disease progression.

## Materials and Methods

### Ethics Statement

This study was carried out in strict accordance with the recommendations made by the Canadian Council for Animal Care. The protocol was approved by the Animal Care Committee at the PMH/OCI institute, protocol number 929.

### Mice and diabetes monitoring

All mice used in the experiments are on the C57BL/6 background. Wild type C57BL/6, p35−/− and p40−/− mice were purchased from the Jackson laboratory [64], [65]. RIP-gp and P14 TCR transgenic mice [36], [39] were maintained in our animal facility according to institutional guidelines. P19−/− and IFNαR−/− mice were kind gifts from N. Ghilardi and D. Pinschewer [66], [67]. Genotyping for all mice were performed by PCR. Diabetes induction was monitored by blood glucose measurements prior to treatment and then 2–3 times per week after treatment. Blood glucose levels were measured using Accu-Chek III glucometers and Chemstrips (Roche).

### LCMV infection

LCMV WE strain was originally obtained from F. Lehmann-Grube [68] and grown on L929 cells and titrated as previously described [69]. RIP-gp/p40+/+ and RIP-gp/p40−/− mice were infected with 3000 PFU of LCMV WE and monitored for alterations in blood glucose.

### BMDC culture

Bone marrow was extracted from the femur and tibia passed through a screen and washed in cold media. 2×10^6^ bone marrow cells were resuspended in 10 ml of DC media (RPMI, 10% LPS free FCS, β-mercaptoethanol, L-glutamine, 40 ng/ml GM-CSF(PeproTech)) and cultured in 10 cm petri dishes at 37°C. On day 3, 10 ml of DC media was added to the culture. On day 6 and 8, 10 ml of culture was removed, resuspended in 10 ml of fresh DC media and added back to the culture. On day 9, BMDCs were resuspended in DC media^−^ (ex GM-CSF) at 2×10^6^cell/ml, re-plated in 24 well plates and stimulated with LPS (100 ng/ml), Poly I∶C (100 µg/ml), and/or IFNα (1000 u/ml). On day 10, BMDCs were pulsed for 2 hours with gp33 peptide alone or gp33, gp276 and gp61 peptides (10^−6^ M) respectively, and washed prior to use in *in vitro* proliferation assays or intravenous infusions into treated mice. For intravenous infusions, 2×10^6^ BMDCs prepared in 200 µl of sterile HBSS were given to each treated mouse. For *in vivo* treatment, mice were infused with 10000 u of IFNα.

### Flow cytometric analyses

BMDCs, cell cultures and single cell suspensions from spleens were stained with antibodies specific for CD11c, CD11b, CD80, CD86, MHCI CD8, CD44, TNF and IL-12p40/p70 flow-cytometry antibodies (BD and eBioscience). Flow cytometry data was acquired on FACSCanto (BD) and anaylzed with Flowjo (Tree Star).

### In vitro proliferation assay

Single cell suspensions were prepared from the spleen of P14 transgenic mice and CD8^+^ T cells were purified using CD8 negative sort kit (Miltenyi Biotech). Purified CD8^+^ T cells were then labeled with CFSE (5-[and-6]-carboxyfluorescein diacetate, succinimidyl ester). 1×10^5^ CFSE-labeled CD8^+^ T cells were co-cultured with 2×10^4^ gp33 peptide pulsed BMDCs in 200 µl of complete IMDM in 96 well round-bottom plates and incubated for 3 days in a 37°C incubator.

### CFSE (5-[and-6]-carboxyfluorescein diacetate, succinimidyl ester) labeling

After washing single cell suspensions in serum-free RPMI 1640 (GIBCO BRL), cells were resuspended in serum-free media at 10^7^ cells per 200 µl containing 10 µM CFSE (Molecular Probes). After incubation for 15 minutes in 37°C incubator, cells were washed in RPMI 1640 containing 10% FCS (Sigma-Aldrich).

### In vivo CTL assay

RIP-gp mice were injected intravenously with 2×10^6^ gp33, gp276 and gp61 peptide-pulsed, LPS/PolyI∶C stimulated BMDCs. 5 days after the initial treatment, single cell suspensions from spleens of C57BL/6 mice were pulsed with gp33 or AV peptide (10^−6^ M) and labeled with CFSE at 1.5 µM and 20 µM respectively. 2×10^7^ each of gp33-pulsed and AV-pulsed CFSE-labeled splenocytes were injected intravenously into each treated RIP-gp mouse. 4 hours later, these mice were sacrificed and single cell suspensions from their spleens were analyzed. The percent specific lysis is calculated by dividing the number of CFSE^Hi^ cells by the number of CFSE^Lo^ cells.

### Intracellular cytokine staining

Intracellular cytokine staining was performed using the BD Cytofix/Cytoperm kit as per manufacturer's instructions.

### Cytometric bead array analysis

Media from BMDC cultures were collected after overnight stimulation with LPS or Poly I∶C. The CBA analysis was performed using the BD CBA assay as per manufacturer's instructions.

### Bone marrow chimeras

Bone marrow was extracted from IFNαR+/+ and IFNαR−/− donor mice previously treated with CD4 and CD8 depleting antibodies. 4×10^6^ bone marrow cells were transferred intravenously into irradiated (9 Grays) sex-matched RIP-gp recipients, and recipients were treated with BMDCs for analysis 6–13 weeks after reconstitution.

### IFNα assay

Media from BMDC cultures were collected after a 12-hour stimulation with LPS or Poly I∶C and assayed with mouse IFN Alpha ELISA kit from PBL Interferon Source, as per manufacturer's instruction.

### Immunohistochemistry

Immunohistochemistry were performed as previously described [70].

## References

[1] Kawai T, Akira S (2007). Signaling to NF-kappaB by Toll-like receptors.. Trends Mol Med.

[2] Iwasaki A, Medzhitov R (2010). Regulation of adaptive immunity by the innate immune system.. Science.

[3] Banchereau J, Pascual V (2006). Type I interferon in systemic lupus erythematosus and other autoimmune diseases.. Immunity.

[4] Blanco P, Palucka AK, Pascual V, Banchereau J (2008). Dendritic cells and cytokines in human inflammatory and autoimmune diseases.. Cytokine Growth Factor Rev.

[5] Garza KM, Chan SM, Suri R, Nguyen LT, Odermatt B (2000). Role of antigen-presenting cells in mediating tolerance and autoimmunity.. J Exp Med.

[6] Lang KS, Recher M, Junt T, Navarini AA, Harris NL (2005). Toll-like receptor engagement converts T-cell autoreactivity into overt autoimmune disease.. Nat Med.

[7] Waldner H, Collins M, Kuchroo VK (2004). Activation of antigen-presenting cells by microbial products breaks self tolerance and induces autoimmune disease.. J Clin Invest.

[8] Eriksson U, Ricci R, Hunziker L, Kurrer MO, Oudit GY (2003). Dendritic cell-induced autoimmune heart failure requires cooperation between adaptive and innate immunity.. Nat Med.

[9] Gonnella PA, Waldner H, Del Nido PJ, McGowan FX (2008). Inhibition of experimental autoimmune myocarditis: peripheral deletion of TcR Vbeta 8.1, 8.2+ CD4+ T cells in TLR-4 deficient mice.. J Autoimmun.

[10] Rifkin IR, Leadbetter EA, Busconi L, Viglianti G, Marshak-Rothstein A (2005). Toll-like receptors, endogenous ligands, and systemic autoimmune disease.. Immunol Rev.

[11] Hirschfeld M, Weis JJ, Toshchakov V, Salkowski CA, Cody MJ (2001). Signaling by toll-like receptor 2 and 4 agonists results in differential gene expression in murine macrophages.. Infect Immun.

[12] Re F, Strominger JL (2001). Toll-like receptor 2 (TLR2) and TLR4 differentially activate human dendritic cells.. J Biol Chem.

[13] Takeuchi O, Hoshino K, Kawai T, Sanjo H, Takada H (1999). Differential roles of TLR2 and TLR4 in recognition of gram-negative and gram-positive bacterial cell wall components.. Immunity.

[14] Hoebe K, Du X, Georgel P, Janssen E, Tabeta K (2003). Identification of Lps2 as a key transducer of MyD88-independent TIR signalling.. Nature.

[15] Yamamoto M, Sato S, Hemmi H, Hoshino K, Kaisho T (2003). Role of adaptor TRIF in the MyD88-independent toll-like receptor signaling pathway.. Science.

[16] Honda K, Yanai H, Negishi H, Asagiri M, Sato M (2005). IRF-7 is the master regulator of type-I interferon-dependent immune responses.. Nature.

[17] Guiducci C, Ott G, Chan JH, Damon E, Calacsan C (2006). Properties regulating the nature of the plasmacytoid dendritic cell response to Toll-like receptor 9 activation.. J Exp Med.

[18] Honda K, Ohba Y, Yanai H, Negishi H, Mizutani T (2005). Spatiotemporal regulation of MyD88-IRF-7 signalling for robust type-I interferon induction.. Nature.

[19] Trinchieri G (2003). Interleukin-12 and the regulation of innate resistance and adaptive immunity.. Nat Rev Immunol.

[20] Ma X, Trinchieri G (2001). Regulation of interleukin-12 production in antigen-presenting cells.. Adv Immunol.

[21] Joffre O, Nolte MA, Sporri R, Reis e Sousa C (2009). Inflammatory signals in dendritic cell activation and the induction of adaptive immunity.. Immunol Rev.

[22] Skokos D, Nussenzweig MC (2007). CD8- DCs induce IL-12-independent Th1 differentiation through Delta 4 Notch-like ligand in response to bacterial LPS.. J Exp Med.

[23] Mescher MF, Curtsinger JM, Agarwal P, Casey KA, Gerner M (2006). Signals required for programming effector and memory development by CD8+ T cells.. Immunol Rev.

[24] Haddad W, Cooper CJ, Zhang Z, Brown JB, Zhu Y (2003). P-selectin and P-selectin glycoprotein ligand 1 are major determinants for Th1 cell recruitment to nonlymphoid effector sites in the intestinal lamina propria.. J Exp Med.

[25] Knippertz I, Hesse A, Schunder T, Kampgen E, Brenner MK (2009). Generation of human dendritic cells that simultaneously secrete IL-12 and have migratory capacity by adenoviral gene transfer of hCD40L in combination with IFN-gamma.. J Immunother.

[26] Su Z, Frye C, Bae KM, Kelley V, Vieweg J (2008). Differentiation of human embryonic stem cells into immunostimulatory dendritic cells under feeder-free culture conditions.. Clin Cancer Res.

[27] Alleva DG, Pavlovich RP, Grant C, Kaser SB, Beller DI (2000). Aberrant macrophage cytokine production is a conserved feature among autoimmune-prone mouse strains: elevated interleukin (IL)-12 and an imbalance in tumor necrosis factor-alpha and IL-10 define a unique cytokine profile in macrophages from young nonobese diabetic mice.. Diabetes.

[28] Liu J, Beller DI (2003). Distinct pathways for NF-kappa B regulation are associated with aberrant macrophage IL-12 production in lupus- and diabetes-prone mouse strains.. J Immunol.

[29] Grabie N, Delfs MW, Westrich JR, Love VA, Stavrakis G (2003). IL-12 is required for differentiation of pathogenic CD8+ T cell effectors that cause myocarditis.. J Clin Invest.

[30] Trembleau S, Penna G, Gregori S, Giarratana N, Adorini L (2003). IL-12 administration accelerates autoimmune diabetes in both wild-type and IFN-gamma-deficient nonobese diabetic mice, revealing pathogenic and protective effects of IL-12-induced IFN-gamma.. J Immunol.

[31] Yang Z, Chen M, Fialkow LB, Ellett JD, Wu R (2003). Inhibition of STAT4 activation by lisofylline is associated with the protection of autoimmune diabetes.. Ann N Y Acad Sci.

[32] Mannon PJ, Fuss IJ, Mayer L, Elson CO, Sandborn WJ (2004). Anti-interleukin-12 antibody for active Crohn's disease.. N Engl J Med.

[33] Murphy CA, Langrish CL, Chen Y, Blumenschein W, McClanahan T (2003). Divergent pro- and antiinflammatory roles for IL-23 and IL-12 in joint autoimmune inflammation.. J Exp Med.

[34] Cua DJ, Sherlock J, Chen Y, Murphy CA, Joyce B (2003). Interleukin-23 rather than interleukin-12 is the critical cytokine for autoimmune inflammation of the brain.. Nature.

[35] Collison LW, Workman CJ, Kuo TT, Boyd K, Wang Y (2007). The inhibitory cytokine IL-35 contributes to regulatory T-cell function.. Nature.

[36] Ohashi PS, Oehen S, Buerki K, Pircher H, Ohashi CT (1991). Ablation of “tolerance” and induction of diabetes by virus infection in viral antigen transgenic mice.. Cell.

[37] Oppmann B, Lesley R, Blom B, Timans JC, Xu Y (2000). Novel p19 protein engages IL-12p40 to form a cytokine, IL-23, with biological activities similar as well as distinct from IL-12.. Immunity.

[38] Mescher MF, Agarwal P, Casey KA, Hammerbeck CD, Xiao Z (2007). Molecular basis for checkpoints in the CD8 T cell response: tolerance versus activation.. Semin Immunol.

[39] Pircher H, Burki K, Lang R, Hengartner H, Zinkernagel RM (1989). Tolerance induction in double specific T-cell receptor transgenic mice varies with antigen.. Nature.

[40] Boonstra A, Rajsbaum R, Holman M, Marques R, Asselin-Paturel C (2006). Macrophages and myeloid dendritic cells, but not plasmacytoid dendritic cells, produce IL-10 in response to MyD88- and TRIF-dependent TLR signals, and TLR-independent signals.. J Immunol.

[41] Akira S, Hemmi H (2003). Recognition of pathogen-associated molecular patterns by TLR family.. Immunol Lett.

[42] Cousens LP, Orange JS, Su HC, Biron CA (1997). Interferon-alpha/beta inhibition of interleukin 12 and interferon-gamma production in vitro and endogenously during viral infection.. Proc Natl Acad Sci U S A.

[43] Aichele P, Unsoeld H, Koschella M, Schweier O, Kalinke U (2006). CD8 T cells specific for lymphocytic choriomeningitis virus require type I IFN receptor for clonal expansion.. J Immunol.

[44] Kolumam GA, Thomas S, Thompson LJ, Sprent J, Murali-Krishna K (2005). Type I interferons act directly on CD8 T cells to allow clonal expansion and memory formation in response to viral infection.. J Exp Med.

[45] Barrat FJ, Coffman RL (2008). Development of TLR inhibitors for the treatment of autoimmune diseases.. Immunol Rev.

[46] Nitta Y, Kawamoto S, Tashiro F, Aihara H, Yoshimoto T (2001). IL-12 plays a pathologic role at the inflammatory loci in the development of diabetes in NOD mice.. J Autoimmun.

[47] Monteiro JM, Harvey C, Trinchieri G (1998). Role of interleukin-12 in primary influenza virus infection.. J Virol.

[48] Orange JS, Biron CA (1996). An absolute and restricted requirement for IL-12 in natural killer cell IFN-gamma production and antiviral defense. Studies of natural killer and T cell responses in contrasting viral infections.. J Immunol.

[49] Schijns VE, Haagmans BL, Wierda CM, Kruithof B, Heijnen IA (1998). Mice lacking IL-12 develop polarized Th1 cells during viral infection.. J Immunol.

[50] Oxenius A, Karrer U, Zinkernagel RM, Hengartner H (1999). IL-12 is not required for induction of type 1 cytokine responses in viral infections.. J Immunol.

[51] Cousens LP, Peterson R, Hsu S, Dorner A, Altman JD (1999). Two roads diverged: interferon alpha/beta- and interleukin 12-mediated pathways in promoting T cell interferon gamma responses during viral infection.. J Exp Med.

[52] Longhi MP, Trumpfheller C, Idoyaga J, Caskey M, Matos I (2009). Dendritic cells require a systemic type I interferon response to mature and induce CD4+ Th1 immunity with poly IC as adjuvant.. J Exp Med.

[53] Blanco P, Palucka AK, Gill M, Pascual V, Banchereau J (2001). Induction of dendritic cell differentiation by IFN-alpha in systemic lupus erythematosus.. Science.

[54] Kono DH, Baccala R, Theofilopoulos AN (2003). Inhibition of lupus by genetic alteration of the interferon-alpha/beta receptor.. Autoimmunity.

[55] Sporri R, Reis e Sousa C (2005). Inflammatory mediators are insufficient for full dendritic cell activation and promote expansion of CD4+ T cell populations lacking helper function.. Nat Immunol.

[56] Devendra D, Jasinski J, Melanitou E, Nakayama M, Li M (2005). Interferon-alpha as a mediator of polyinosinic:polycytidylic acid-induced type 1 diabetes.. Diabetes.

[57] Lang KS, Georgiev P, Recher M, Navarini AA, Bergthaler A (2006). Immunoprivileged status of the liver is controlled by Toll-like receptor 3 signaling.. J Clin Invest.

[58] Lang PA, Cervantes-Barragan L, Verschoor A, Navarini AA, Recher M (2009). Hematopoietic cell-derived interferon controls viral replication and virus-induced disease.. Blood.

[59] Jiang A, Bloom O, Ono S, Cui W, Unternaehrer J (2007). Disruption of E-cadherin-mediated adhesion induces a functionally distinct pathway of dendritic cell maturation.. Immunity.

[60] Menges M, Rossner S, Voigtlander C, Schindler H, Kukutsch NA (2002). Repetitive injections of dendritic cells matured with tumor necrosis factor alpha induce antigen-specific protection of mice from autoimmunity.. J Exp Med.

[61] Wakkach A, Fournier N, Brun V, Breittmayer JP, Cottrez F (2003). Characterization of dendritic cells that induce tolerance and T regulatory 1 cell differentiation in vivo.. Immunity.

[62] Reis e Sousa C (2006). Dendritic cells in a mature age.. Nat Rev Immunol.

[63] Kaka AS, Foster AE, Weiss HL, Rooney CM, Leen AM (2008). Using dendritic cell maturation and IL-12 producing capacity as markers of function: a cautionary tale.. J Immunother.

[64] Magram J, Connaughton SE, Warrier RR, Carvajal DM, Wu CY (1996). IL-12-deficient mice are defective in IFN gamma production and type 1 cytokine responses.. Immunity.

[65] Mattner F, Magram J, Ferrante J, Launois P, Di Padova K (1996). Genetically resistant mice lacking interleukin-12 are susceptible to infection with Leishmania major and mount a polarized Th2 cell response.. Eur J Immunol.

[66] Ghilardi N, Kljavin N, Chen Q, Lucas S, Gurney AL (2004). Compromised humoral and delayed-type hypersensitivity responses in IL-23-deficient mice.. J Immunol.

[67] Muller U, Steinhoff U, Reis LF, Hemmi S, Pavlovic J (1994). Functional role of type I and type II interferons in antiviral defense.. Science.

[68] Weidt G, Deppert W, Utermohlen O, Heukeshoven J, Lehmann-Grube F (1995). Emergence of virus escape mutants after immunization with epitope vaccine.. J Virol.

[69] Battegay M, Cooper S, Althage A, Banziger J, Hengartner H (1991). Quantification of lymphocytic choriomeningitis virus with an immunological focus assay in 24- or 96-well plates.. J Virol Methods.

[70] Nguyen LT, Elford AR, Murakami K, Garza KM, Schoenberger SP (2002). Tumor growth enhances cross-presentation leading to limited T cell activation without tolerance.. J Exp Med.

